# Autologous Cord Blood Therapy for Infantile Cerebral Palsy: From Bench to Bedside

**DOI:** 10.1155/2014/976321

**Published:** 2014-02-20

**Authors:** A. Jensen

**Affiliations:** Campus Clinic Gynecology, Ruhr-University Bochum, Universitätsstraße 140, 44799 Bochum, Germany

## Abstract

About 17 million people worldwide live with cerebral palsy, the most common disability in childhood, with hypoxic-ischemic encephalopathy, preterm birth, and low birth weight being the most important risk factors. This review will focus on recent developments in cell therapy for infantile cerebral palsy by transplantation of autologous umbilical cord blood. There are only 4 publications available at present; however, the observations made along with experimental data *in vivo* and *in vitro* may be of utmost importance clinically, so that a review at an early developmental stage of this new therapeutic concept seems justified. Particularly, since the first published double-blind randomized placebo-controlled trial in a paradigm using allogeneic cord blood and erythropoietin to treat cerebral palsy under immunosuppression showed beneficial therapeutic effects in infantile cerebral palsy, long-held doubts about the efficacy of this new cell therapy are dispelled and a revision of therapeutic views upon an ailment, for which there is no cure at present, is warranted. Hence, this review will summarize the available information on autologous cord blood therapy for cerebral palsy and that on the relevant experimental work as far as potential mechanisms and modes of action are concerned.

## 1. Introduction

About 17 million people worldwide live with cerebral palsy, the most common disability in childhood, with hypoxic-ischemic encephalopathy, preterm birth, and low birth weight being the most important risk factors [1, 2]. Among 142 surviving very low birth weight infants at a corrected age of 2 years, there were 23.2% minor and 25.4% severe disabilities [3], from the latter of which 1 in 2 will die before the age of 18 years. Furthermore, a metaanalysis revealed that among children with cerebral palsy, 3 in 4 were in pain; 1 in 2 had an intellectual disability; 1 in 3 could not walk; 1 in 4 could not talk; 1 in 4 had epilepsy; and 1 in 10 were blind [2]. This reflects the magnitude of the personal, medical, and socioeconomic burden of this devastating brain disorder [4]. In the United States the estimated lifetime costs to society for treatment and care for persons born in 2000 with cerebral palsy amount to US$ 11.5 billion [5]. Thus, causative treatments along with definition of responsive brain disorders, timing of intervention, and definition of risk groups are needed to prevent the sequelae of infantile cerebral palsy.

This review will focus on recent developments in cell therapy for infantile cerebral palsy by transplantation of autologous umbilical cord blood because therapeutic strategies using cord blood in other human neurologic entities, including those for lysosomal storage disorders [6], have recently been reviewed elsewhere [7–10]. There are only 4 publications on autologous cell therapy available at present; however, the observations made along with experimental data *in vivo *and *in vitro *may be of utmost importance clinically, so that a review at an early developmental stage of this new therapeutic concept seems justified. Particularly, since the first published double-blind randomized placebo-controlled trial in a paradigm using allogeneic cord blood and erythropoietin to treat cerebral palsy under imunosuppression showed beneficial therapeutic effects in infantile cerebral palsy [11]. Thus, long-held doubts about the efficacy of this new cell therapy, that were in part related to the “homing” mechanism, are dispelled and a revision of therapeutic views upon an ailment, for which there is no cure at present, is warranted.

However, transplantation of allogeneic cord blood for cerebral palsy is limited by immunologic responses of the host (host-versus-graft reaction) and therefore requires immunosuppression, which in turn causes both inflammatory and neurotoxic side effects as well as reduced therapeutic efficacy depending on the degree of HLA mismatch [11, 12]. Hence, this review will summarize only the available information on autologous cord blood therapy for cerebral palsy and that on the relevant experimental work as far as potential mechanisms and modes of action are concerned.

## 2. Preclinical Studies

### 2.1. Human Umbilical Cord Blood Cells “Home” and Prevent Spastic Paresis

**Figure 1:**
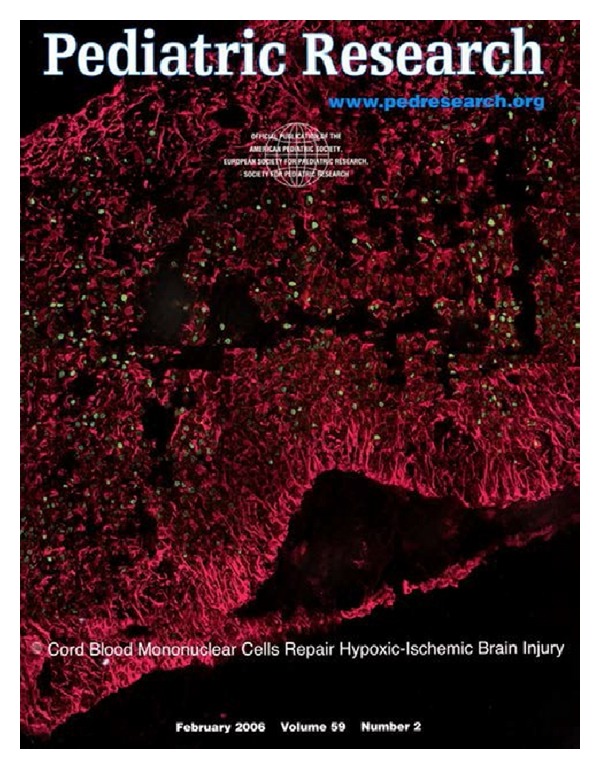
Intraperitoneal transplantation of mononuclear hUCB-derived cells resulted in a specific “homing” of these cells into the CNS and incorporation around the lesioned area. Title page from [17, Figure  3(B)]. HLA-DR-positive mononuclear cells (*green*) are located within a scaffold of GFAP-positive astrocytes (*red*) in the area of the hypoxic-ischemic lesion. This paper is available online at: http://www.nature.com/pr/journal/v59/n2/full/pr200649a.html.

**Figure 2:**
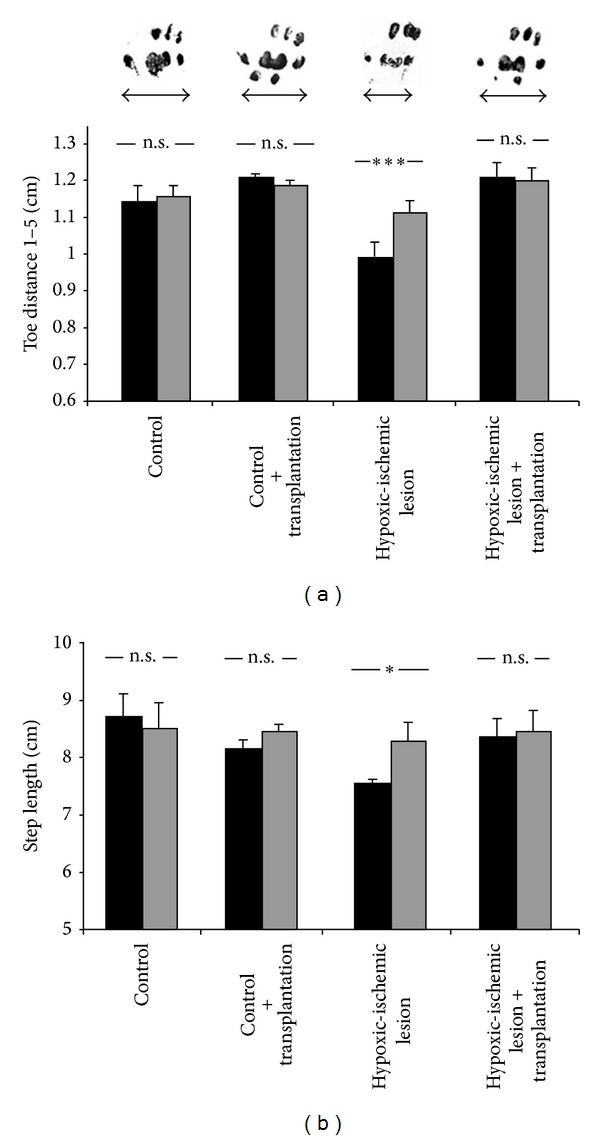
Transplantation of hUCB-derived mononuclear cells reduces spastic paresis as assessed by footprint analysis of 3-wk-old animals (see [17, Figure  5]). (a) Hypoxic-ischemic brain damage results in spastic paresis of the distal limb muscles, causing a significant reduction of footprint width (toe distance 1 to 5) of the right hind paw (contralateral to the insult; *black columns*) compared with the left (ipsilateral; *gray columns*) hind paw. Intraperitoneal transplantation of hUCB-derived mononuclear cells after hypoxic-ischemic brain damage reduced spastic paresis. In these animals, differences between ipsi- and contralateral hind paws were no longer detectable. Photographs of footprints (right hind paws) illustrate the footprint widths (*arrows*) of control animals without (*left*) and with (*center left*) transplantation, upon hypoxic-ischemic lesion without (*center right*) and with (*right*) transplantation of hUCB-derived mononuclear cells. (b) In control animals with and without transplantation, the step length of left and right hind paws is equal. In contrast, hypoxic-ischemic lesion resulted in a significantly reduced step length of the right hind paw (*black columns*) compared with the left hind paw (*gray columns*). This reduction in step length of the hind paw contralateral to the lesion, also indicative of spastic paresis, was largely alleviated upon transplantation of hUCB-derived mononuclear cells. Data are presented as mean ± SEM; *P < 0.05; ***P < 0.001. This paper is available online at: http://www.nature.com/pr/journal/v59/n2/full/pr200649a.html.

First mention of the concept of amelioration of perinatal brain damage by neuroregeneration using cord blood dates back to 2002, when our preliminary data in chronically prepared fetal sheep showed a highly specific invasion of human umbilical cord blood mononuclear cells (huMNC) into the damaged brain region, that is, “homing,” in a model of cerebral ischemia [13]. This was the beginning of a series of experiments *in vitro *and *in vivo*, using a different animal model (“Levine”) of perinatal cerebral hypoxic-ischemia in newborn rats to increase effectiveness [14, 15]. The “Levine” model includes unilateral carotid artery ligation followed by 8% O_2_ inhalation for 80 minutes on postnatal day (PN) 7. The neurodevelopment of the rat at PN 7 is equivalent to that of the human brain at birth [16]. These studies yielded three major insights. First, the confirmation that unmanipulated huMNC transplanted systemically 24 hours after the insult invade the damaged brain area in large numbers in a highly specific manner (“homing”), as observed in the sheep model [13]. Unlike in the latter (transplantation into the umbilical vein), in the rat model we had chosen to transplant the mononuclear cells intraperitoneally [14], thus providing the unique opportunity to observe that these cells migrate long distance to the damaged area when appropriate signals are released [17] (Figure 1). Secondly, this “homing” of huMNC was strictly confined to hypoxic-ischemic regions showing microglial activation, assessed immunohistochemically by CD68 expression, along with signs of apoptotic activity, assessed by cleaved caspase-3 expression. Not one single huMNC was found elsewhere in the brain. There is growing evidence that among others inflammatory-mediating effects and mesenchymal stem cells are involved [18]. Thirdly, and most importantly, we observed that huMNC transplanted systemically after cerebral hypoxic-ischemia prevented the development of spastic paresis [17] (Figure 2). If to whether this involved prevention and/or restoration of white matter damage and how oligodendrocytes might be protected, remains to be established [19, 20]. It is important to notice that these effects were demonstrated in an acute treatment paradigm transplanting huMNC shortly after the insult. There is, however, no information as yet on the efficacy of a delayed treatment and how this may rate limit the effects of cells on the development of the functional connectome [21].

### 2.2. Cord Blood Cells Release Growth Factors, Cytokines, and Chemokines

**Figure 3:**
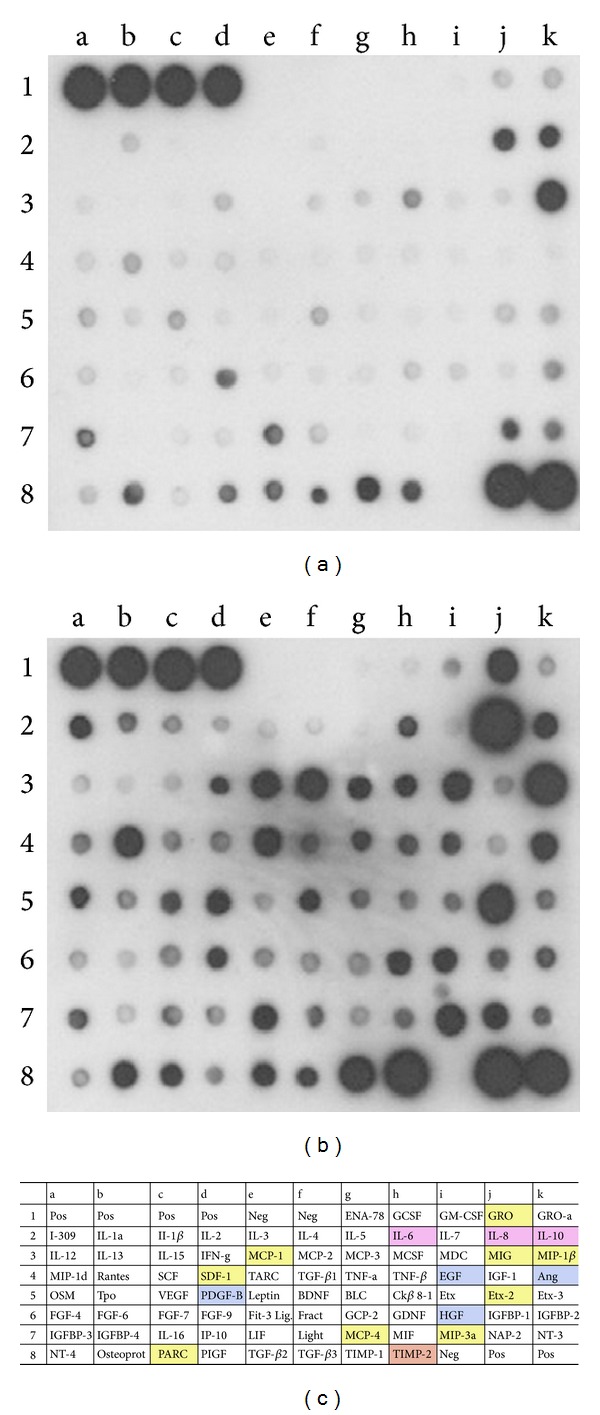
Protein antibody array detecting cytokines in human umbilical cord blood (hUCB)-cell conditioned media (see [22, Figure  7]). Representative examples of two antibody array membranes, incubated with either nonconditioned culture medium (a) or culture medium conditioned by hUCB-derived mononuclear cells for 2 days (b). Increasing intensity reveals increased secretion of the cytokines investigated. Dots a1 to d1 and j8 to k8 were positive controls (Pos); dots e1 to f1 and i8 were negative controls (Neg). (c) Overview of all proteins assayed on the membrane. Boxed factors are color-coded and refer to those proteins secreted at significant levels. Colors designate interleukin proteins (purple), growth factors (blue), chemokines (yellow), as well as tissue inhibitors of metalloproteinase2 (TIMP-2) (orange). Ang: angiogenin; BDNF: brain-derived neurotrophic factor; BLC: B-lymphocyte chemoattractant; EGF: epidermal growth factor; ENA-78: epithelial neutrophil-activating protein-78; Etx; eotaxin; FGF; fibroblast growth factor; Fract: fractalkine; GCP-2: granulocyte chemotactic protein-2; GCSF: granulocyte colony-stimulating factor; GDNF: glial cell-derived neurotrophic factor; GM-CSF: granulocyte macrophage colony-stimulating factor; GRO: growth-regulated oncogene; HGF: hepatocyte growth factor; IFN-g: interferon-g; IGF-1: insulin-like growth factor-1; IGFBP: insulin-like growth factor binding protein; IL: interleukin; IP-10: interferon-inducible protein-10; LIF: leukemia inhibitory factor; MCP: monocyte chemoattractant protein; MCSF: macrophage colony-stimulating factor; MDC: macrophage derived chemokine; MIF: macrophage inhibitory factor; MIG: monokine induced by g-interferon; MIP: macrophage inflammatory protein; NAP-2: neutrophil activating protein-2; NT: neurotrophin; OSM: oncostatin M; Osteoprot: osteoprotegrin; PARC: pulmonary and activation-regulated chemokine; PDGF-B: platelet-derived growth factor-B; PIGF: placenta growth factor; SCF: stem cell factor; SDF-1: stromal-derived factor-1; TARC: thymus associated and regulated chemokine; TGF-b: transforming growth factor-b; TNF: tumor necrosis factor; TIMP: tissue inhibitor of melloproteinases; Tpo: thrombopoietin; VEGF: vascular endothelial growth factor.

Studies *in vitro *revealed that among others a number of growth factors, anti-inflammatory cytokines, and chemokines are released from huMNC upon stimulation (Figure 3) [22]. This supported the concept that the “homing” of these cells into the damaged brain area may exert indirect beneficial effects rather than replace neurones, for example, by transdifferentiation, as it was speculated in the first place. In fact, in our hands, unlike in others also using rats [23, 24], there was no evidence for transdifferentiation. However, the most intriguing finding beyond “homing” was that in newborn rats transplantation of huMNC after cerebral hypoxic-ischemia prevented spastic paresis, the key symptom of cerebral palsy in animals as well as in humans [17]. This effect was independent of the site of transplantation, that is, intraperitoneal or intrathecal [25], but the mechanisms involved in functional neuroregeneration are unknown.

### 2.3. The Chemokine SDF-1 Promotes “Homing” 

As far as mechanisms and modes of action are concerned, chemokines promoting “homing” are of major interest [26]. Among others, stromal derived factor (SDF)-1, expressed in the damaged brain region, plays a significant role by binding to the CXCR4-receptor on huMNC. This in turn elicits long distance migration of these cells to the damaged brain region. The finding that in newborn rats SDF-1 is a major chemokine involved in this mechanism is supported by receptor blocking experiments using monoclonal antibodies that showed prevention of “homing” to a large extent [26]. The residual “homing” observed may well be caused by different chemokines released from huMNC [22], for example, MIP-1 alpha or MCP-1 (Figure 3), as shown in the adult rat stroke model [27]. However, there are also other mechanisms to consider.

### 2.4. Cord Blood Cells Reduce Glial Scars and Restore Cortical Neural Processing

**Figure 4:**
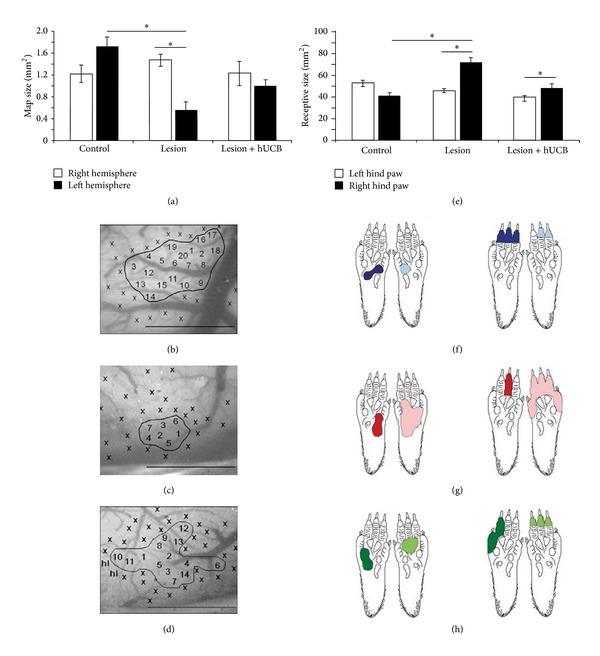
Effects of hypoxic ischemic brain injury and hUCB treatment on receptive field (RF) and cortical map size (see [28]). (a) In lesioned rats the size of the left cortical hindpaw (HP) representation was significantly reduced after hypoxic ischemic brain injury (HI) (*P* = 0.005 versus controls, *P* = 0.004 versus contralateral hemisphere). Treatment with hUCB cells prevented map changes in the left cortical HP representation. (b)–(d) images of the cortical surface of the left hemisphere of a control (b), lesioned (c), and hUCB treated rat (d). Numbers indicate penetration sites; x indicates noncutaneous responses. hl: hindlimb. Borders of the maps are outlined. Scale bar 1 mm. (e) In lesioned rats RF size of the right HP was increased (*P* = 0.007 versus controls, *P* = 0.03 versus HP ipsilateral to the lesion). hUCB treatment leads to moderate RF increase, not significantly different from controls (*P* = 0.558). Bars represent sem (f)–(h) examples of RFs on the right and left HP for a control (f), lesioned (g), and hUCB treated rat (h). Number of rats used: control group *n* = 10, lesion group *n* = 17, and hUCB group *n* = 6. A total of 975 RFs were recorded (left hemisphere: control 127: lesioned 97, treated 85; right hemisphere: control 192, lesioned 327, and treated 147). doi: 10.1371/journal.pone.0020194.g003. This paper is available online at: http://www.plosone.org/article/info:doi/10.1371/journal.pone.0020194.

As shown in a recent study on cerebral hypoxic-ischemia in newborn rats, transplanted huMNC cause a reduced glial activation along with reduced gap junction proteins (CX43), thus reducing glial scar formation [25] so that neuroregenerative processes may be entrained to such an extent that even neuroprocessing in the primary somatosensory cortex recovers [28]. Specifically, it has been shown in the same model that the dimensions of cortical maps and receptive fields, which were significantly altered after brain damage, were largely restored, and hyperexcitability was no longer observed in huMNC treated animals, as indicated by a paired-pulse behavior, resembling that observed in control animals [28]. The beneficial effects on cortical processing were reflected in an almost complete recovery of sensorimotor behavior. Thus, huMNC reinstall the way central neurons process information by normalizing inhibitory and excitatory processes [28] (Figure 4).

But not only the somatosensory motor behavior, also gross and fine motor function as well as muscle strength were improved upon transplantation of huMNC after hypoxic-ischemic brain damage in newborn rats both medium and long term [25]. These beneficial effects are independent of the transplantation site. This holds also true for the fore limb use bias in the long term, an index to describe the lateralization of the limb use towards the healthy side after hypoxic-ischemia during erection of the animals in a perspex cylinder. Interestingly, there was a clear time course of recovery, in that, unlike spasticity [17], the fore limb bias was still significantly different from control at 2 weeks (P21), but not at 6 weeks (P51) after transplantation [25].

Glial cells play a crucial role in neuroregeneration, specifically, activation of astrocytes as part of the inflammatory response may be involved in the formation of glial scars that limit neuronal plasticity after brain damage in newborn rats [25]. This response also led to accumulation of activated microglia/macrophages as indicated by increased CD68 (ED1) expression, a lysosomal protein and a member of the LAMP4 family, allowing “homing” of macrophage subsets to particular sites where the tissue damage is most pronounced (http://www.genecards.org/cgi-bin/carddisp.pl?gene=CD68). Interestingly, the CD68 (ED1) immunosignals in brain regions representing cortical motor areas for fore and hind limbs were significantly smaller in transplanted animals, an effect that was long-lasting (6 weeks) and irrespective of transplantation site of huMNC [25, 29], suggesting that the blood brain barrier had no influence on these effects in this model. In line with a reduced activation of microglia/macrophages, there was also a reduced astrocytic glial fibrillary acidic protein (GFAP) immunoreactivity, suggesting both diminished inflammatory response and glial activation after transplantation [25].

**Figure 5:**
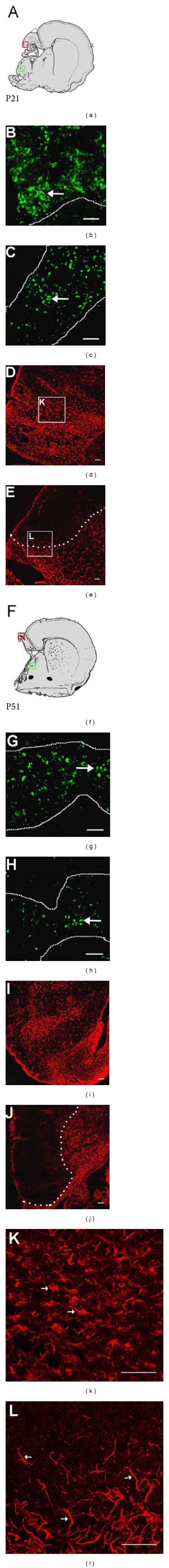
Reduction of microglial infiltration and GFAP expression after hUCB transplantation (see [25]). Schematic drawings show representative coronal brain sections after hypoxic-ischemic lesion corresponding to Bregma *þ*1.2-(*þ*) 0.2 mm at P21 (a) and P51 (f). Boxes highlight photographed areas (green: CD68 expression in the basal ganglia; red: GFAP expression in the cortex). (b) HI led to an infiltration of CD68 positive microglia. Cells were present at the lesion site throughout the late acute ischemic (P21) and, to a lesser extent, in the chronic postischemic phase ((g) P51). Dotted lines delineate histological defined areas of prominent tissue damage, and these express higher levels of CD68. Long white arrows indicate clusters of CD68 positive cells. Brains of transplanted animals showed a reduced microglial infiltration at both time points compared to nontransplanted animals ((c) P21; (h) P51). In analogy, two weeks following HI a massive upregulation of GFAP encircling the lesion site was observed (d) and maintained until seven weeks of age (i). hUCB transplantation led to a reduced amount of GFAP immunoreactivity around the ischemic zone ((e) P21; (j) P51), thus leaving a larger portion of the remaining cortex spare of the inflammatory reaction ((e) and (j) white dotted line indicates border of cortical areas with lower versus those with higher GFAP expression). Astrocytes in the vicinity of the ischemic zone presented an activated phenotype characterized by a large soma and fewer processes, thus obtaining a more rounded shape ((k) white arrows). In hUCB transplanted animals, a higher number of astrocytes appeared to have a more elongated phenotype with long processes ((l) white arrows). Scale bars: 50 *μ*m. (For interpretation of the references to color in this figure legend, the reader is referred to the web version of this paper.) This paper is available online at: http://www.sciencedirect.com/science/article/pii/S0006899312011511.

The distribution of gap junction protein CX43 reflected that of GFAP, while a zone of upregulation was observed in the inner glial wall of astrocytes and microglia, which separated the lesion core from the surrounding parenchyma. Again there was a reduced CX43 expression after huMNC transplantation, suggestive of less glial scar formation. The observed changes were quantified by Western blot analysis and quantitative real-time PCR of GFAP and CX43 on proteins and mRNA at 1 day (P9), 2 weeks (P21), and 6 weeks (P51) after transplantation, revealing already after 1 day a downregulation of astrocytic protein and mRNA [25] (Figure 5). These results were confirmed by quantitative analysis of src-homologous phosphotyrosine phosphatase 1 (SHP-1) expression, a protein known to be associated with reduced astroglial proliferation. Unlike in animals after hypoxic-ischemia without treatment, in transplanted animals SHP-1 was reduced to control levels after initial elevation [30, 31].

In summary, transplantation of huMNC reduces glial scar formation and improves motor deficits medium and long term after hypoxic-ischemia in the newborn rat. This is paralleled by reduced infiltration of activated microglia/macrophages and decreased expression of GFAP, CX43, and SHP-1 mRNA and protein, all of which are known to increase after hypoxic-ischemia [32–34]. Importantly, reduced scar formation might be a prerequisite of functional neuroregeneration, that is in part mediated by T cells in the umbilical blood [35, 36].

### 2.5. Neuronal Survival

The mechanisms by which transplanted huMNC exert functional recovery in the brain are largely unknown. Hence, studies on the cellular and molecular level are required. Recently, using our model of hypoxic-ischemia in newborn rats [17], the effects of huMNC transplantation on apoptosis, the number of vital neurons, expression of neurotrophic and angiogenic factors, and on the integrity of the blood brain barrier have been examined [37]. Specifically, cleaved caspase-3 and neuronal nuclei protein Neu-N expression, markers for apoptotic cell death, have been quantified using immunohistochemistry and immunoblot analysis. There was clear evidence for improved neuronal survival caused by huMNC transplantation after brain injury.

Furthermore, expression of brain-derived neurotrophic factor (BDNF) and vascular endothelial growth factor (VEGF) revealed already two days after the insult that both growth factors, known to inhibit apoptosis and inflammation (BDNF) and to promote vasodilatation, angiogenesis, and neuroprotection (VEGF), declined in untreated but showed no change (BDNF) or an increase (VEGF) in transplanted animals [37]. Thus, both growth factors may contribute to neuroprotection [38] by neurotrophic and proangiogenic action [39].

### 2.6. Blood Brain Barrier

In our first set of experiments on transplantation of huMNC after hypoxic-ischemic brain damage in newborn rats, we hypothesized that—unlike in control animals that received a transplantation without brain lesion—the blood brain barrier must have been at least transiently destroyed [17] to explain the “homing” of huMNC into the damaged brain area in large numbers [40]. This would lead to a “facilitated intrusion of MNC and a preferential regional incorporation of these cells” [17]. To test this hypothesis, the changes in expression of tight junction protein occludin, a marker for blood brain barrier integrity, and angiopoietin receptor tyrosine-kinase with immunoglobulin-like and EGF-like domains (Tie)-2, an endothelial protein associated with angiogenesis, were studied immunohistochemically and by immunoblot analysis [37]. There was clear evidence for a breakdown of the blood brain barrier, indicated by decreased expression of occludin two days after the insult (P9), followed by an increase of occludin to control levels at 2 weeks (P21), suggesting molecular reconstruction of the blood brain barrier at this point. Similarly, there was clear evidence of increased expression of Tie-2 protein levels after transplantation of huMNC, suggesting vascular regeneration and angiogenesis after the insult by the transplanted cells [37, 41]. Thus, the proposed hypothesis of transient breakdown of the blood brain barrier along with vascular cerebral impairment as a basis for the “homing” process has been supported [17]. However, it has not been tested if the breakdown of the blood brain barrier is essential for “homing” or a coincidental event. Further, the fact of reconstruction of the blood brain barrier at two weeks after the insult is potentially at variance with clinical observations showing improved outcome after (very) delayed treatment. This raises the important issue of whether human umbilical cord blood mononuclear (stem) cells can cross the intact blood brain barrier.

## 3. Autologous Cord Blood Transplantation in Infantile Cerebral Palsy

**Figure 6:**
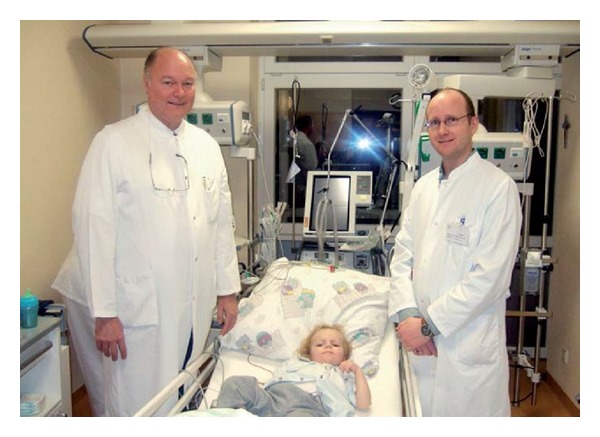
First autologous cord blood transplantation after global hypoxic-ischemic brain damage caused by cardiac arrest in a boy 2.5 years of age (see [43]). Nine weeks after the insult the patient (L. B.) received an autologous cord blood transplantation to treat cerebral palsy (January 27, 2009). The boy was normally developed when brain damage occurred, that was followed by a quadriplegic persistent vegetative state (see video S3 in Supplementary Material [44]). From left to right: A. Jensen, M. D.; patient L. B.; E. Hamelmann, M. D., Ruhr-University Bochum, Germany. This paper is available at: http://www.campus-klinik-bochum.de/pdf/Jensenrm12011.pdf.

**Figure 7:**
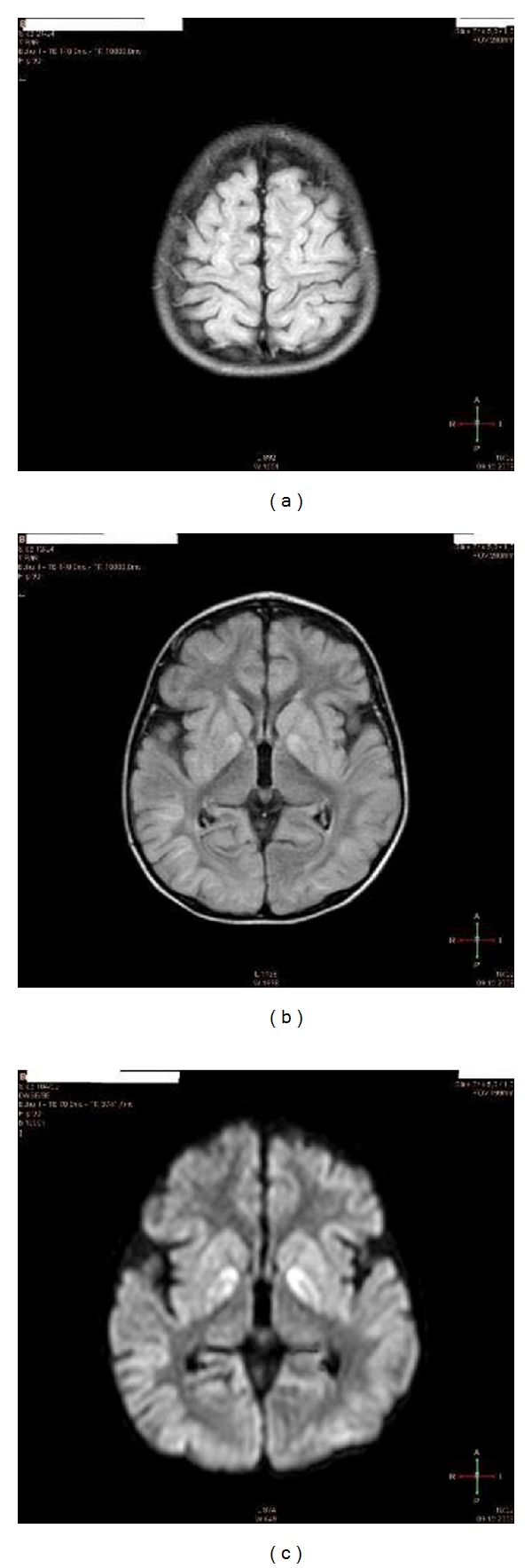
Brain MRI of the patient (L. B.) 2 weeks after cardiac arrest. Note, signs of severe global ischemia in cortical structures as evidenced by (a) signal hyperintensity of gyri in almost entire cortex (FLAIR sequences) and basal ganglia (b), including caudate nucleus and putamen (c) (FLAIR DWI sequences with contrast media) (see video S1, S2 in Supplementary Material [44]).

**Figure 8:**
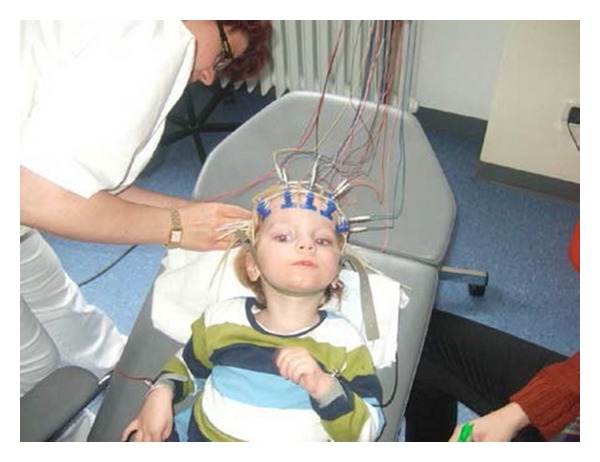
EEG recording (L. B.) before transplantation. EEG recording. The patient (L. B.) is in a persistent vegetative state 9 weeks after the insult before transplantation of cord blood cells. Note, the dilated, unresponsive pupils in spite of bright light from the ceiling (see video S3 in Supplementary Material [44]).

**Figure 9:**
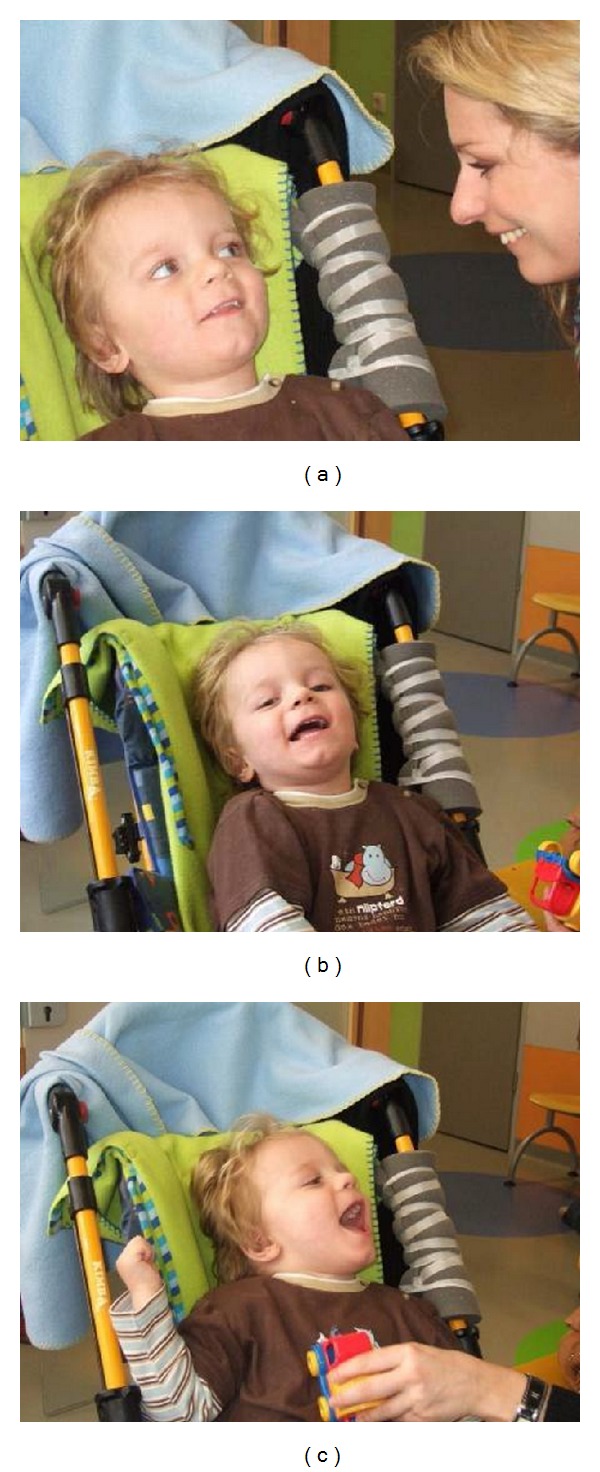
Two-month follow-up: (a) first social smiling of the patient (L. B.) towards his mother and (b), (c) laughing, when played with, 2 months after autologous transplantation of cord blood cells (i.e., 4 months and one week after severe brain damage caused by cardiac arrest). (see video S4 in Supplementary Material [44]).

**Figure 10:**
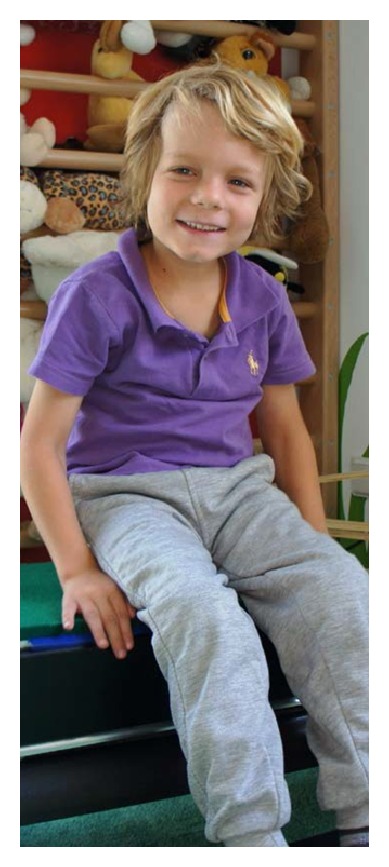
4.5-year follow-up. The boy (L. B.) has now entered primary school at the age of 7 and 4.5 years after transplantation of autologous cord blood after global hypoxic-ischemic brain damage caused by cardiac arrest followed by a quadriplegic persistent vegetative state. He is still using a posterior gait trainer for ambulation. (Follow-up at 2 years see video S5, S6 in Supplementary Material [44]).

Clinical data on autologous cord blood transplantation for treatment of cerebral palsy are scarce [42–45] and have largely been collected for safety and feasibility reasons. There is only one videodocumented case of autologous cord blood transplantation after severe global hypoxic-ischemic brain damage caused by cardiac arrest, leaving the 2.5-year old boy in a quadriplegic persistent vegetative state [43, 44] (Figure 6). Accordingly, MRI revealed signal hyperintensity in both the entire cortex and basal ganglia (Figures 7(a), 7(b), and 7(c)) (see also video S1, S2 in Supplementary Material [44]), and EEG rhythm and frequency were grossly disturbed showing transient isoelectric traces. On the day of transplantation, 9 weeks after the insult, the boy presented quadriplegic cerebral palsy (see video S3) [44]; his pupils were still dilated showing only minimal response to direct light (Figure 8). He was blind (cortical blindness), deaf, and whimpered permanently. One week after transplantation he stopped whimpering, at 4 weeks he executed simple tasks on demand, motor control improved, and spastic paresis was largely reduced [43]. At 7 weeks, EEG was normal and eyesight recovered in part; he started smiling when played with (Figures 9(a), 9(b), and 9(c)) and was able to sit with support and to speak simple words (Ma-ma, Pa-pa) (see video documentation, S4) [44]. At 1 year after transplantation, spastic paresis was further reduced free sitting and walking with support were possible. After 2 years, there was independent eating, crawling, passive standing, and walking in a gait trainer (see video documentation S5) [44]. Fine motor control had improved to such an extent that the boy managed to steer a remote control car (see video documentation S6) [44]. After 3 years, receptive and expressive speech competence also improved significantly (four-word sentences, 200 words), and there was broad understanding [44]. Now, at the age of 7 and 4.5 years after transplantation, the boy has entered primary school (Figure 10), though still using a posterior gait trainer for ambulation (Crocodile Retrowalker).

Papadopoulos and coworkers first reported on 2 toddlers with diagnosed cerebral palsy that received a combination of autologous cord blood, low dose subcutaneous granulocyte colony stimulating factor (G-CSF) injection before and/or after cord blood transfusion, and hyperbaric oxygen therapy [42]. Though there were no abnormalities in MRI imaging before and after treatment and both experienced uneventful deliveries at term by caesarean section with normal Apgar scores and weights without obvious signs of obstetrical risk factors, the toddlers presented spastic diplegia and were unable to walk at 19 months (case 1) and 15 months (case 2), respectively, the latter only being able to stand briefly using orthosis. Both toddlers were classified as level III in the Gross Motor Function Classification System (GMFCS). In both cases significant improvements in motor ability were noted at 7 weeks and at 36 months after cord blood transfusion with reduced spasticity bilaterally, allowing them to walk almost independently, to run (case 1), and to swim. In both cases reclassification to GMFCS level I was possible [42].

The largest cohort of autologous cord blood transplantation for cerebral palsy has been published by Lee and coworkers [45], who reported on an uncontrolled single arm pilot study of 20 children with cerebral palsy to assess the safety and feasibility of the procedure as well as its potential treatment efficacy (see Table 1, [45]). In this carefully documented study, employing an array of neurodevelopmental examinations and imaging techniques, including brain perfusion SPECT analysis and MRI-DTI (FA values) before and during follow-up, there were overall neurologic improvements in 5/20 children, ranging from 23 to 91 months of age, weighing 7.2–21.4 kilogram (Table 1) [45]. Interestingly, the type of cerebral palsy exhibited mattered, in that patients with diplegia or hemiplegia showed improvements rather than those with quadriplegia.

From these 5 children, MRI FA values were evaluated to assess white matter integrity in 26 regions of interest (ROI). In 3 regions (temporal right, corpus callosum, and right posterior periventricular white matter) significant changes after treatment were noted when compared with pretreatment values. The authors conclude that intravenous cord blood infusion seems to be practical and safe and has yielded potential benefits in children with cerebral palsy [45].

**Figure 11:**
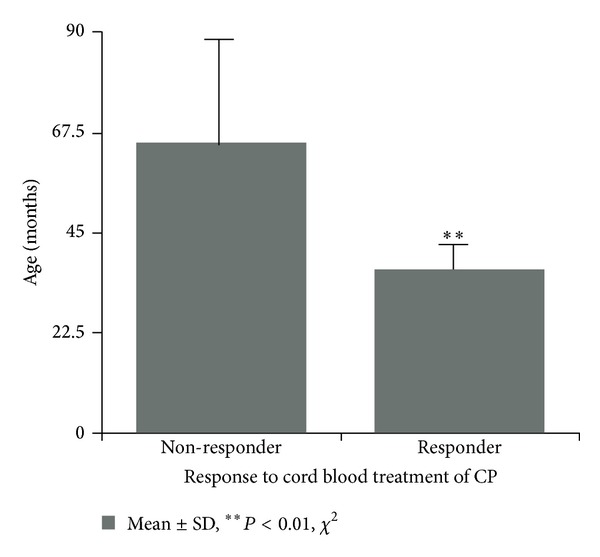
The success of autologous cord blood treatment in children with CP caused by hypoxic-ischemic encephalopathy depends on age. Recalculated from Lee et al., Table 1 [45], (HIE, 8/20, 65.2 ± 23.1 SD months, *n* = 5 versus 37.3 ± 6.0 SD months, *n* = 3, **P < 0.01, Chi-Square-test). The paper from which the data for recalculation were derived is available online at: http://www.ncbi.nlm.nih.gov/pmc/articles/PMC3369209/.

Interestingly, though age at treatment did not show any significant differences in global outcome (*n* = 20), it is noteworthy that within the subgroup of hypoxic-ischemic encephalopathy (HIE, *n* = 8), the non-responders were significantly older (65.2 ± 23.1 SD months, *n* = 5) than the responders who showed improvements after treatment (Figure 11) (37.3 ± 6.0 SD months, *n* = 3, *P* < 0.01, Chi-Square-test, recalculated from Table 1, [45]). Also, it is conspicuous from Table 1 that in this study the majority (15/20) of conditions possibly related to cerebral palsy did not respond to treatment. Future clinical trials, which are on the way [10], will have to define clearly which patient will profit and when treatment should be provided for optimum effect.

### 3.1. Safety Aspects

The children treated for cerebral palsy by intravenous autologous cord blood transplantations only experienced mild adverse effects, including transient hemoglobinuria, nausea, and hypertension in one case [44], mild transient nausea in 2 cases [42], hemoglobinuria plus nausea in 3/20 cases, and hemoglobinuria plus urticaria in 2/20 additional cases [45].

The largest retrospective study on safety and feasibility of intravenous infusion of autologous cord blood, in which 184 children with acquired neurologic disorders received 198 cord blood infusions (there are no neurological outcome data available from this study), reports on only 3 (1,5%) patients showing adverse effects [46]. These patients presented infusion reactions, all responding to medical therapy and stopping the infusion. All cord blood units were thawed and washed in dextran-albumine, and premedication with acetaminophen, diphenhydramine, and methylprednisolone was provided. Interestingly, these authors report on postthaw sterility cultures that were positive in 7.6% of the infused cord blood units without causing infections in the patients, though antibiotics were not given. Intravenous infusions lasted 15–20 minutes and iv-fluids were administered at twice maintenance for 2 to 4 hours after cord blood infusion.

Vital signs and pulse-oximetry were monitored every 5 minutes during infusion and every 30 minutes for 2 to 4 hours after infusion [46]. Thus, on the basis of the available information, autologous cord blood transfusion can be considered a safe and feasible treatment. However, we have to bear in mind that the effective dose and timing of administration are not known and side effects are almost always dose dependent.

### 3.2. Future Challenges

In order to avoid giving false hopes to patients and their families, it will be of utmost importance for ongoing and future clinical trials [10] to define the groups of brain disorders responding to autologous cord blood transplantation and also to define the respective therapeutic windows to provide the optimum timing for treatment of infantile cerebral palsy. Conversely, to maximize the benefit for responders, risk groups need to be defined, such as children presenting hypoxic-ischemic encephalopathy and/or very low birth weight infants showing evidence of brain damage, for example, periventricular leukomalacia. Also, sophisticated logistics have to be developed to provide effective treatment “on-site” shortly after the insult to prevent rather than to treat cerebral palsy, perhaps combined with hypothermic neuroprotection. This proposed ambitious “on-site” emergency treatment paradigm is a worthwhile challenge for tertiary perinatal centers, which, above all, requires a change in cord blood banking policy, in that autologous cord blood banking becomes the rule rather than the exception. Once implemented, this new and causative treatment option offers the historic opportunity for obstetricians, neonatologists, and neuropediatricians to combat infantile cerebral palsy effectively for the first time.

## 4. Conclusions

From an experimental point of view, treatment for acute hypoxic-ischemic brain damage using mononuclear cells from human umbilical cord blood was shown to be effective, at least in the model that we and others employed. Also, insights in some important aspects regarding potential mechanisms and modes of action involved have been gathered. However, since there is virtually no information neither on the optimum amount of cells needed nor on the ideal timing of transplantation after the insult to be effective, future work should also focus on these clinically important questions.

From a clinical point of view, intravenous autologous cord blood therapy for infantile cerebral palsy is safe, however, based on the preliminary uncontrolled clinical data available, it appears only to be effective in certain cases. It is important to notice that in the majority of uncontrolled cases it was not effective. Future work and ongoing clinical trials primarily must define both the groups of brain disorders responding and the therapeutic window, in which autologous cord blood therapy may be effective, in order to avoid giving false hopes to the patients and their families. Time matters—in several respects.

## Figures and Tables

**Table 1:** Therapeutic responses to autologous cord blood infusion according to clinical characteristics and infused TNC counts. This paper is available online at: http://www.ncbi.nlm.nih.gov/pmc/articles/PMC3369209/ [45].

UPN	Sex	Age (Mo)	BW (Kg)	TNC (10^7^/kg)	Dx			Tx response				
					Type	GA	Possible causes	DDST II	PEDI	GMFCS	MACS	Overall
1	M	80	13	5.38	Quad	Full	Unknown	N	Y	N	N	N
2	F	24	10	6.72	Quad	Full	HIE, PVL	N	N	Y	N	N
3	F	91	14	10.92	Quad	Full	Unknown	N	Y	N	N	N
4	F	91	19.5	2.87	Quad	Full	HIE, MAS	N	N	N	N	N
5	F	82	18.1	4.36	Di	Full	Unknown	N	Y	N	N	N
6	F	28	7.2	9.58	Quad	Full	Unknown	N	N	N	N	N
7	M	31	11.2	5.72	Quad	Full	Strep meningitis	N	N	N	N	N
8	F	71	21.4	2.89	Hemi	Full	Polymicrogyria	Y	Y	N	N	Y
9	M	43	16.4	0.6	Hemi	Preterm	HIE, PVL	Y	Y	N	Y	Y
10	F	75	9.4	10.63	Quad	Full	HIE, MAS	N	N	N	N	N
11	F	53	15.3	5.88	Hemi	Full	Unknown	N	Y	N	N	Y
12	F	40	12.7	2.44	Hemi	Preterm	HIE, PVL	N	Y	N	Y	Y
13	M	29	10.8	6.85	Di	Preterm	HIE, PVL	Y	Y	N	N	Y
14	M	67	15	5.26	Quad	Full	Unknown	N	N	N	N	N
15	M	78	20	5.14	Hemi	Full	HIE, ICH, Infarction	Y	Y	N	N	N
16	F	71	17.9	2.86	Di	Full	Unknown	N	Y	N	N	N
17	F	58	11.6	15.65	Quad	Preterm	HIE, PVL	N	Y	N	N	N
18	M	29	11.3	0.71	Quad	Full	Unknown	N	Y	N	N	N
19	F	30	11.5	3.29	Hemi	Full	MCA infarction	N	N	N	N	N
20	M	23	10.9	2.25	Quad	Full	Unknown	N	Y	N	N	N

UPN: unique patient number, BW: body weight, TNC: total nucleated cells, Dx: diagnosis, Tx: treatment, GA: gestational age, DDST  II: Denver developmental screening test II, PEDI: pediatric evaluation of disability inventory, GMFCS: Gross Motor Function Classification System, MACS: Manual Ability Classification System, Quad: quadriplegic, Di: diplegic, Hemi: hemiplegic, Full: full term, HIE: hypoxic ischemic encephalopathy, PVL: periventricular leukomalacia, MAS: meconium aspiration pneumonia, Strep: streptococcal, ICH: intracranial hemorrhage, MCA: middle cerebral artery, N: no, and Y: yes.
